# Art perception is affected by negative knowledge about famous and unknown artists

**DOI:** 10.1038/s41598-024-58697-1

**Published:** 2024-04-07

**Authors:** Hannah Kaube, Rasha Abdel Rahman

**Affiliations:** Department of Psychology, Neurocognitive Psychology, Humboldt-Universität zu Berlin, Berlin, Germany

**Keywords:** Human behaviour, Object vision, Perception, Emotion

## Abstract

The biographies of some celebrated artists are marked by accounts that paint a far from beautiful portrait. Does this negative-social knowledge influence the aesthetic experience of an artwork? Does an artist’s fame protect their paintings from such an influence? We present two preregistered experiments examining the effect of social–emotional biographical knowledge about famous and unknown artists on the reception and perception of their paintings, using aesthetic ratings and neurocognitive measures. In Experiment 1, paintings attributed to artists characterised by negative biographical information were liked less, evoked greater feelings of arousal and were judged lower in terms of quality, than paintings by artists associated with neutral information. No modulation of artist renown was found. Experiment 2 fully replicated these behavioural results and revealed that paintings by artists associated with negative social-emotional knowledge also elicited enhanced early brain activity related to visual perception (P1) and early emotional arousal (early posterior negativity; EPN). Together, the findings suggest that negative knowledge about famous artists can shape not only explicit aesthetic evaluations, but may also penetrate the perception of the artwork itself.

## Introduction

The surrealist maestro Salvador Dalí can be credited for some of the most iconic and highly acclaimed artworks of the 20th Century. On the other side of the canvas, he has admitted to beating an admirer until she was bloody, assaulting a blind amputee, and having violent fantasies about hurting several women^1^. Indeed, history is rife with examples of famous artists made infamous through their actions, rhetoric or beliefs. Emerging evidence suggests that negative information about (unnamed) artists can influence aesthetic outcomes and underlying emotional processes^2^; however, the influence of social–emotional biographical knowledge about famous artists has yet to be studied systematically. To this end, we analysed aesthetic judgments and electrophysiological brain responses associated with the perception, evaluation, and emotional arousal evoked by artworks attributed to both well-known and unknown artists, whilst orthogonally manipulating the valence of the biographical information. By additionally investigating the renown of the artists and including real, well-known painters, we aimed to replicate and extend the previous research, while emphasising relevance and external validity of the experimental content. Further, by exploring the potential interaction between fame and valence of knowledge, we sought to explore whether an artist’s fame serves as a shield, protecting their art from the impact of negative information, or renders it more vulnerable to such effects.

An artwork is seen as a conduit of meaning and intention between artist and art recipient^3,4^ and is generally regarded as a corporeal extension of its creator^5^. Accordingly, a growing body of research highlights the influence that artist-related information can exert on an aesthetic experience, beyond the physical characteristics of the work itself^6–14^. While some types of information, such as the amount of time spent on an artwork^15^, may be readily discerned when looking at it, other types of artist-related information, which are not directly related to a specific piece of work (e.g., an artist’s eccentricity^9^, nationality^13^, and disability^16^), have also been found to influence different aesthetic outcomes. Such findings are consistent with theoretical aesthetic models which stress the importance of the interplay between the visual properties of an image and various contextual factors, including knowledge^17–19^. Neuroscientific studies also indicate that artist-related contextual information is integrated during art perception and activates higher-order cognitive functions^6,10^. For example, activation in the medial orbitofrontal cortex, a brain region associated with cognitive processes such as reward representation, demonstrates a greater association with aesthetic judgments when the artwork being viewed is presented as human-made, compared to computer generated^10^. Overall, the research shows that different types of information can affect the processing of an artwork at different stages, resulting in a diverse pattern of aesthetic outcomes^18^. It also suggests that our experience of an artwork may not be entirely separable from knowledge about the artist, although some facets of the experience may be more susceptible to influence than others^2^.

An artist’s renown serves as a further source of artist-related information which can affect aesthetic judgments. Research indicates that the same painting is liked more, found more beautiful and judged as more interesting when it is attributed to a famous, rather than non-famous artist^20^. Similarly, art is evaluated differently across a number of dimensions (e.g., quality, artist talent, emotional value) depending on whether it is labelled as having been painted by a famous artist or a copyist^21,22^. The reverence of artists who have “passed the test of time” has been shown to be strengthened and maintained via repeated exposure^23^. Indeed, informing participants that an artwork is not by a famous artist reduces the well-established correlation between familiarity and liking^18,24^. Beyond exposure and familiarity, works by renowned artists are often ascribed greater aesthetic and cultural merit^25^ and an artist’s fame contributes to the cultural and economic worth of their paintings in the art market^26,27^. This wealth of literature emphasises the importance of considering fame when investigating the influence of an artist’s biography on art perception.

Turning to the impact of social-emotional biographical knowledge, the link between artist and artwork is less direct and not as well-researched. A recent study explicitly investigating the influence of negative-social (vs neutral) information about artists reported effects on aesthetic evaluations and neurocognitive measures^2^. Participants learnt either neutral or negative socially relevant information about the artists of a series of paintings, and rated each image for liking, arousal and quality. Images allegedly painted by “bad” artists were liked less and found more arousing than those allegedly painted by artists associated with neutral information. Electrophysiological brain responses, extracted from the EEG, revealed an enhanced early posterior negativity (EPN); a component associated with the fast, reflexive and arousal-related processing of emotionally valenced visual stimuli^28–30^. Later, more elaborate and controlled evaluation was not affected by biographical information, as indexed by the lack of an effect in a later positive potential (LPP)^31–33^. It was concluded that aesthetic outcomes related to emotion are particularly sensitive to social-emotional knowledge about artists and that such knowledge is integrated rapidly during early stages of stimuli processing. Although insightful, the study’s findings cannot be extrapolated onto works by all artists. Real-world instances of an artist’s moral transgression would generally only be widely reported or of interest if a large amount of people were familiar with the artist. Moreover, the presented information did not refer to any of the artists by name; the resulting anonymity may further reduce the relevance and ecological validity of the experimental content.

**Figure 1:**
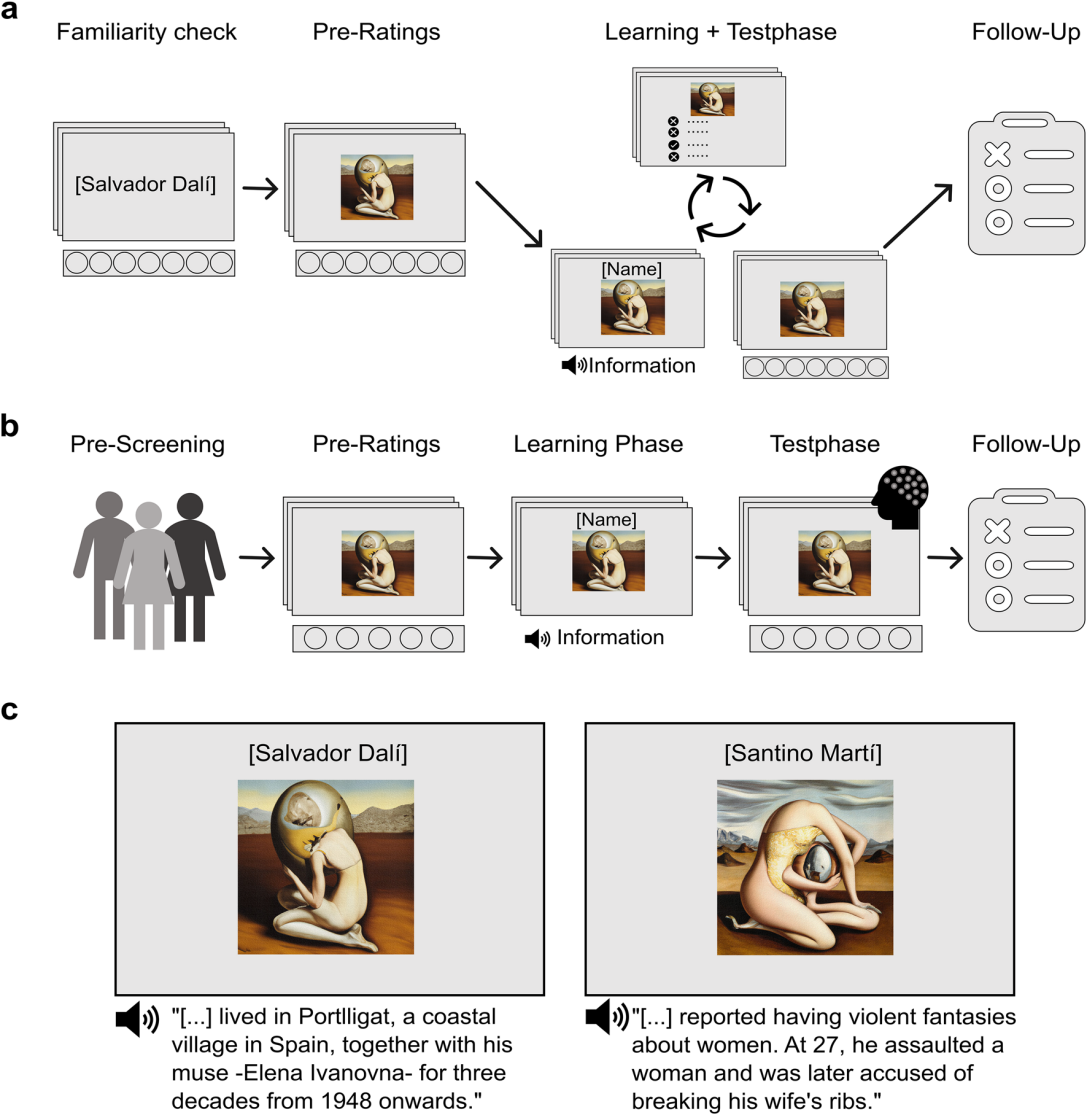
Experimental paradigm. (**a**) Experiment 1 procedure overview. Participants were first required to indicate how familiar they were with the famous and unknown artists’ names (exclusion criteria). Paintings were then rated for liking, arousal and quality. The learning and test phases were combined and split into smaller blocks, such that information was learnt about the artists of 5 paintings at a time, followed by an attention check and the post-learning ratings. Covariates were obtained in a follow-up questionnaire at the end. (**b**) Experiment 2 procedure overview. A pre-screening was conducted to ensure that only participants familiar with the famous artists took part in the main experiment. All paintings were rated for liking, arousal and quality, before and after knowledge acquisition. EEG was recorded during the post-learning liking ratings. Interest and willingness to display a painting were also assessed. The post-experiment questionnaire measured the ability to recall the information and several covariates. (**c**) Schematic illustration of the experimental manipulation. An unknown counterpart was constructed for each famous artist. In the learning phase, participants were presented with the paintings whilst simultaneously hearing negative or neutral information about the artists. The information always started with an artist’s name. The names were also presented above each painting. To exclude a possible confound of image familiarity, none of the presented paintings were created by well-known artists. Paintings were matched for content, style and complexity and selected on the basis of representing the famous artist’s style. The assignment of images to conditions (negative-famous, neutral-famous, negative-unknown, neutral-unknown) was fully counterbalanced across participants. In the experiment, original artworks from different artists were used. Images have been created by the authors using the OpenAI software DALLE-2 for publication purposes. Sentences have been translated from German.

To address these issues, we adapted the design of the aforementioned study^2^ to include the factor fame and conducted a behavioural, online experiment (Experiment 1) and an EEG experiment (Experiment 2). We systematically varied the valence of the artist-related information (social-negative vs social-neutral) as well as participants’ familiarity with the artists (famous vs unknown). In both experiments, participants rated a series of paintings for liking, arousal and quality (see Fig. 1). In Experiment 2, we collected two additional behavioural measures post knowledge acquisition: how interesting participants found each painting and how willing they were to display the painting in their own home. We expected paintings presented in the negative biographical condition to be liked less and rated as more arousing than paintings by artists associated with neutral biographical information^2,34^. We further assumed that evaluations of quality would be made independently of the affective knowledge^2^. Due to the instrumental role of artist renown outlined above, we also hypothesised that paintings by famous artists would be liked more, found more arousing and receive higher judgments of quality than paintings by unknown artists. Crucially, we expected that artist renown would modulate the knowledge effects on the dimensions liking and arousal. As it is plausible that an artist’s fame could protect their works from the effects of negative information, but also conceivable that emotional reactions are more pronounced for works by artists one already knows, the directions of the proposed interactions were formulated as alternative hypotheses. With reference to the additional behavioural measures obtained in the EEG experiment, we expected negative, compared to neutral biographical information to evoke greater interest^34,35^ and render participants less willing to display a painting in their home^36,37^.

In Experiment 2, electrophysiological brain responses from the EEG were also recorded, delineating the electrocortical underpinnings of the knowledge effects at various stages of perceptual and emotional processing. Our primary analyses focused on two ERP components which have been reliably linked to the electrocortical processing of emotional stimuli: early posterior negativity (EPN) and late positive potential (LPP)^28,30–33,38^. Whilst the former is associated with early arousal-related processing, the latter reflects more elaborate evaluations of motivationally relevant emotional stimuli. We expected to replicate the finding that social-negative knowledge about an artist elicits an enhanced EPN amplitude^2^. In contrast to the previous study, artist names were also provided within the information and presented alongside paintings in the present study. Assuming that the provision of an artist’s name increases the social relevance of the stimuli, we hypothesised a knowledge effect on LPP amplitude^39,40^. As the emotional processes reflected in the EPN and LPP component have been shown to be enhanced when negative information is provided about a famous (or familiar), compared to an unfamiliar face^41,42^, we expected the hypothesised knowledge effects to be more pronounced when the information pertained to a famous artist. To further illuminate the time-course of affective knowledge and/or fame effects on perceptual processing, we also analysed P1 and N1/N170 components. These are linked to early stages of low and high-level visual perception^43,44^ and have previously been shown to be modulated by various types of declarative knowledge^45–48^. Lastly, to explore whether the type of information we provided also elicits changes in the semantic representation of the images, the N400, a component associated with the processing of meaning^49^ was also analysed.

## Experiment 1

### Sample size

To assess the required sample size, an a priori power analysis based on the data from Kaube et al.^2^ was calculated using the SIMR package in R^50^. The fixed effect of negative compared to neutral knowledge on liking ratings was estimated. Random intercepts were modelled for participants and items, and random slopes were modelled for the predictor. The resulting effect size (*b* =  − 0.36) was specified in simulations (1000 iterations) of different potential sample sizes, all of which were multiples of 4, to accommodate the perimeters of our balanced design. Results indicated that a sample of 36 would be sufficient to detect an effect with an expected power of 92.5%; 95% CI [90.69, 94.06].

### Participants

Participants were recruited using a university-based recruitment platform. One person did not complete the experiment (data not analysed). None of the participants were excluded for having a formal education in art or art history. All participants passed 75% or more of the attention checks (see “Procedure” section). A total of 15 datasets had to be excluded and replaced because participants either failed to meet the inclusion criteria of not being familiar enough with the famous artists (i.e. less than 6 out of 8 famous artists were given a familiarity rating < 4 on a 7-point scale; N = 11); claimed to know the made-up artists too well (i.e. more than 2 out of 8 made-up names were given a familiarity rating of > 3; N = 3) or both (N = 1). It should be noted that the preregistered requirements of a participant rating the familiarity of at least 7 out of 8 famous artists ≥ 4 and rating the familiarity of a maximum of 1 out of 8 unknown artists ≤ 2 proved more conservative than initially expected and was adjusted accordingly as described above.

The final sample of participants (N = 36; 26 female, 1 diverse; mean age = 30.86, range = 20–45) included only native German speakers with normal (or corrected-to-normal) eyesight and normal colour vision. All individuals provided informed consent before participating and received monetary compensation or credit points for their participation. The study was approved by the local ethics committee and complied with the standards set by the Declaration of Helsinki.

### Materials

Based on a separate online rating study of German native speakers (N = 25; 13 women; mean age = 28.08, range 18–48) eight famous artists were selected to be included in the study. For each famous artist, an “unknown” pendant was created. These were devised in such a way, that the country of origin and the number of syllables in all parts of the name matched their renowned counterparts (e.g., Salvador Dalí/Santino Martí). For each pair of artists, a negative and a neutral story was constructed, yielding a total of four conditions per artist set (famous-neutral, famous-negative, unknown-neutral, unknown-negative). The stories were formulated according to several criteria: (1) each started with the artist’s name, while word length was maintained within a narrow range (± 1 word) within a given set; (2) the information provided was unrelated to the artist’s abilities, reputation, or artistic style; (3) both neutral and negative information was derived from the renowned artist’s biography; however, (4) neither contained explicit details that would overtly connect it to the famous artist, thus ensuring plausibility for the unknown condition as well. For instance, instead of using the well-known pseudonym “Gala”, Salvador Dalí’s muse-turned-wife was referred to by her lesser-known birth name “Elena Diakonova”. Finally, (5) the non-emotional parts of the content referred to in the stories was kept comparable in the neutral and negative versions (e.g., [abusing] a woman vs [living with] a woman).

For each artist pair, two corresponding paintings were selected as visual stimuli. These paintings were matched in terms of content, style and complexity by the authors (see^2^). To control for effects of familiarity, none of the paintings included in the study were created by the named famous artists. However, both paintings of a pair closely resembled the style and artistic era typically associated with the corresponding famous artist, making it plausible that either could have been painted by them. The resulting feeling of familiarity of the paintings (assessed at the end of Experiment 1 on a scale ranging from 1 “definitely never seen the image before today” to 7 “definitely seen the image before today”) was modest: *mean* = 3.50, *sd* = 2.22. To increase the credibility of the selection of images and artists, four highly familiar paintings were included as fillers (e.g., “the Birth of Venus” by Sandro Botticelli) and positive information was created for the respective artists. The dimensions of the matched images were edited to ensure uniformity and the presentation size and resolution across the whole selection was standardised (175,000 pixels with 72 × 72 ppi).

### Procedure

In order to ensure unimpeded audio for the experiment, participants were initially required to correctly type a word that was audibly played via speaker. In the first task of the experiment, each artist name was presented on the screen, with the question “how familiar are you with this name?” underneath it (7-point scale from “not at all” to “very much”). The resultant ratings were used as part of the exclusion criteria (see above). During the pre-learning phase, each painting was presented onscreen for 3.5 s, before being replaced by one of the three rating scales (liking, arousal, quality; see^2^). The rating tasks were conducted block-wise, the order of which was randomised across participants, but kept the same within a participant throughout the duration of the experiment. Within a rating block, the first presented painting was a filler, the order of the rest of the paintings was randomised. Participants were instructed to answer spontaneously and responses were not subjected to a time limit.

Adapted from a well-stablished learning paradigm^2,41,42,51,52^, the learning and test phases were combined and split into smaller blocks (see Fig. 1a). Each block contained five images in total: one from each of the four conditions and a filler. The assignment of images to conditions was fully counterbalanced across participants. All blocks consisted of a learning phase, an attention check and post-ratings (i.e., test phase). First, each painting was presented on-screen with the name of the artist above it while the corresponding biographical information was played simultaneously. After 11 s, the name was replaced by the question “to what extent is the information reflected in the painting?” and a 7-point scale (from “not at all” to “very much”) appeared under the image. The question was designed to ensure that participants were paying attention to the information, without priming them to focus on differences in informational valence. After completing the learning segment, participants were shown one of the five images assigned to the block again and were required to answer a single-choice attention check. Failure to correctly answer 75% of the attention checks was pre-defined as an exclusion criterium. Lastly, the post-ratings were conducted in the same way as specified in the pre-learning phase for the five paintings assigned to a block. For each dependent variable, 288 trials per condition were analysed.

After the main part of the experiment, several other measurements were obtained in a follow-up questionnaire: level of art interest (adapted from^53^); degree of agreement with the statement “I believe one should separate art from the artist”; and a retrospective assessment of personal strategy used to make judgments (answer options: “spontaneous judgments” vs “deliberate effort to ignore information” vs “deliberate effort to integrate information”). These additional variables are not included in further analyses in the present study. At the end of the experiment, participants were fully debriefed as to the aim and nature of the study. The explanation included a statement about the fact that the presented information had been collated from various readily available sources pertaining to the famous artist of a pair (e.g., news articles, biographies, third-party diary entries) and had in part been framed particularly negatively or oversimplified for the purposes of the study. During the debriefing, all participants were also made aware of the fact that the shown images were not created by the named artists and that the information provided did not apply to the real artists of the presented works. The mean duration of the experiment was 29 min.

### Analyses

Statistical analyses were conducted in R using linear mixed effects models (LMMs) at single-trial level. Models were calculated using the lme4 package^54^ and p-values were calculated using the lmerTest package^55^. For each dependent variable (liking, arousal and quality), valence of knowledge (negative vs neutral), artist renown (famous vs unknown) and the interaction between the two factors were specified as fixed effects. To allow for a comparison between spontaneous baseline ratings and the post-learning ratings, these fixed effect terms were nested inside the time of rating (before knowledge acquisition vs after knowledge acquisition). Random intercepts were modelled for participants and items. Aiming to fit the maximum plausible random effects structure^56^, we also modelled random slopes for all the predictors (i.e., knowledge, artist renown, time of rating) and their interactions. Random slopes which prevented model convergence were calculated via singular value decomposition and removed.

### Results

**Figure 2:**
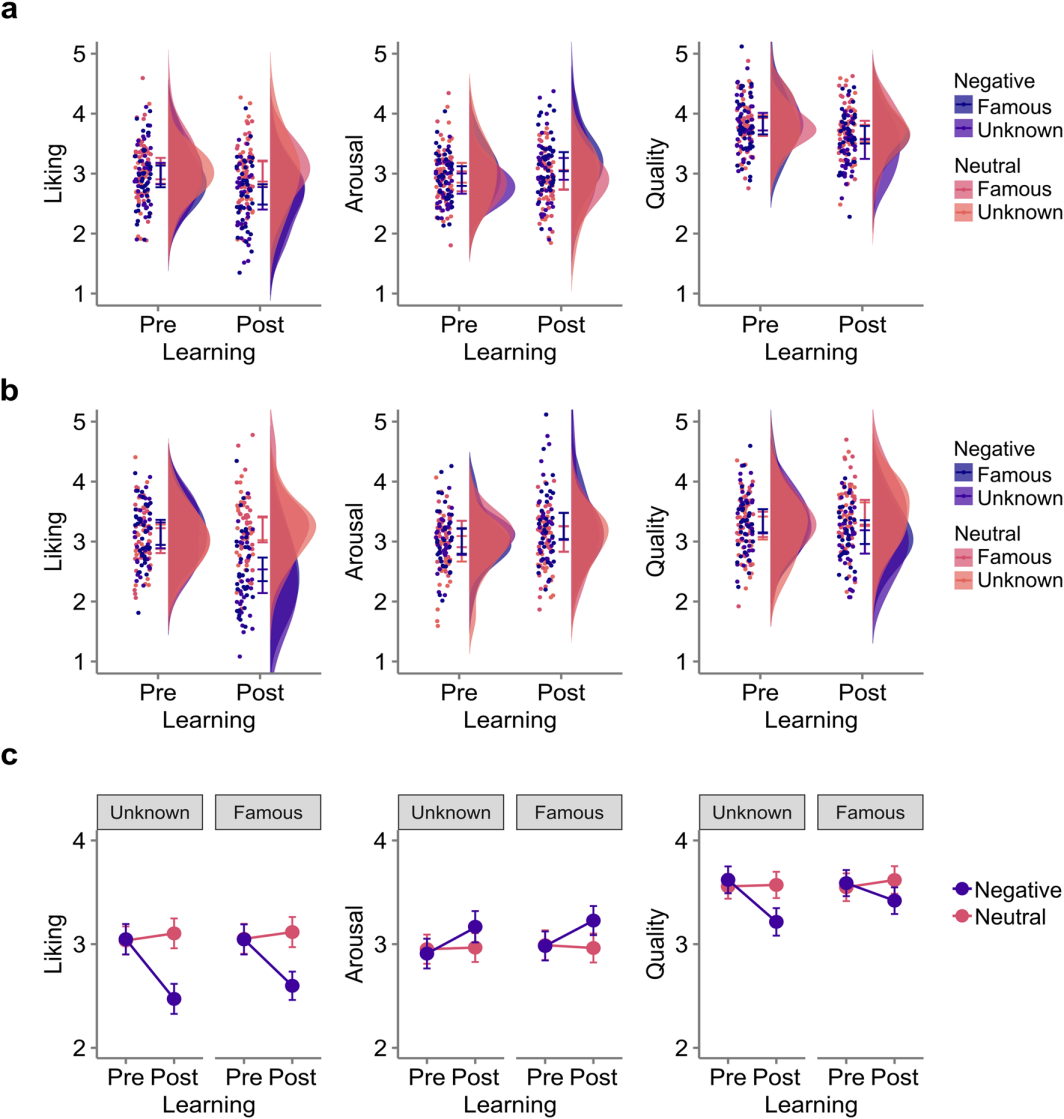
Ratings of liking, arousal and quality before and after acquisition of knowledge. Results from Experiment 1 (**a**) and Experiment 2 (**b**). Raincloud plots show estimations of the probability density as well as data points and 95% confidence intervals aggregated by subject. Higher numbers reflect more liking, greater arousal and greater judgments of quality. In Experiment 1, range of scales was 1 to 7; presented scales have been adjusted via linear transformation for better comparability with the results from Experiment 2. (**c**) Results from pooled analyses. Ratings were z-transformed prior to analyses. Error bars represent 95% confidence intervals.

As illustrated in Fig. 2a, prior to learning affective biographical information, there was no significant difference between conditions for any of the three behavioural dimensions (see Supplementary Table S1 for full statistical output). After the learning phase, a main effect of valence of knowledge was found, such that paintings by artists associated with negative information were liked less, (*b* =  − 0.61, 95% *CI* [− 0.85 to − 0.37], *p* < 0.001), found more arousing (*b* = 0.36, 95% *CI* [0.12 to 0.61], *p* = 0.004) and were judged qualitatively worse (*b* =  − 0.26, 95% *CI* [− 0.44 to − 0.08], *p* = 0.006), than paintings by artists characterised by neutral biographical information. The factor artist renown did not significantly affect liking (*b* = 0.07, 95% *CI* [− 0.17 to 0.31], *p* = 0.554) or arousal ratings (*b* = 0.09, 95% *CI* [− 0.20 to 0.38], *p* = 0.554), but judgments of quality were slightly higher for famous, as opposed to unknown artists (near-significant trend: *b* = 0.23, 95% *CI* [− 0.03 to 0.48], *p* = 0.077). Valence of knowledge and artist renown did not interactively affect any of the aesthetic ratings in these models (liking: *b* = 0.05, 95% CI [− 0.81 to 0.90], *p* = 0.907; arousal: *b* = 0.19, 95% *CI* [− 0.48 to 0.85], *p* = 0.571; quality: *b* = 0.27, 95% *CI* [− 0.22 to 0.76],* p* = 0.277).

## Experiment 2

### Sample size and pre-screening

Based on effects observed in previous studies^2,41,51^ with similar experimental designs and factors of interest, as well as counterbalancing requirements, we preregistered and collected 32 valid data sets. An online pre-screening was created to ensure that only non-experts who were familiar with the selection of famous artists would take part in the main EEG experiment. The names of the artists were interspersed with other famous names from different fields (i.e., science, music, and politics). Participants were asked to classify the occupations of the famous people. An alternative option: “I don’t know” was also provided to minimize guessing as a strategy. At the end, participants were asked to indicate whether they had ever studied art or art history (or science, music or politics as distractor questions). Incorrect classification of more than two famous painters and/or confirmation of a formal background in art were exclusion criteria for the main study. Out of the 69 individuals who participated in the pre-screening, 20 people failed the classification task (29%) and none of the remaining 49 individuals had studied art or art history.

### Participants

The final sample consisted of 32 (mean age = 26.13; range of 18–37; 18 women) right-handed, native German speakers with normal (or corrected-to-normal) eyesight and normal colour vision. One person had to be excluded and replaced due to a technical error during EEG recording and two due to bad quality EEG data. No participants had to be excluded for incorrectly classifying the valence of artist-related information in the follow-up survey (92.6% correct classification; range 68.8–100%). Participants received monetary compensation or credit points. Before participating, all individuals provided informed consent. The study was approved by the local ethics committee and complied with the standards set by the Declaration of Helsinki.

### Materials

The same stimuli and information were used as specified in Experiment 1. The dimensions of the images were adjusted (new total area = 56,400 pixels) to reduce eye-movements during the EEG recording. Viewing distance was set at 70 cm. Visual angles ranged from 4.82° to 7.01° horizontally and from 4.65° to 6.75° vertically, depending on the image pair.

### Procedure

The same experimental procedure was used as in Kaube et al.^2^ (see Fig. 2b). Participants were first required to rate each image for liking on a 5-point scale via button-press. Each trial consisted of a fixation cross, presented for 0.5 s, followed by a painting, which remained onscreen until a participant responded or for a maximum of 2.5 s. The first image presented was consistently a filler, the order of the rest of the paintings was randomised. Participants then rated each image for arousal and quality in the same manner, with the order of the arousal and quality task blocks counterbalanced across participants.

The learning and test phases were constructed in line with previous studies^2,41,42,51,52^, and conducted separately. During the learning phase, participants were simultaneously presented with an image, the artist’s name and the associated information a total of 4 times. Learning blocks were split into smaller mini-blocks which increased in size to ease the acquisition of knowledge. Again, all participants saw all of the paintings and heard all of the stories, but the assignment of images to conditions was fully counterbalanced across participants. In the first part of the test phase, each post-learning liking trial was repeated a total of 16 times while the EEG was recorded (yielding 256 trials per person or 8192 in total). In the second part, post-learning quality and arousal ratings were measured using the identical trial structure described for the pre-learning ratings. Finally, ratings for the two additional dependent behavioural variables “interest” (“not interesting” to “very interesting”) and “willingness to display” (“definitely would not” to “definitely would”) were obtained in counterbalanced blocks.

After completing the experiment, participants took part in a short follow-up questionnaire to ensure that the presented information had been adequately learnt. They were presented with all of the paintings a final time and instructed to correctly classify whether the information they had learnt about the associated artist was neutral or negative (a pre-defined exclusion criterium required correct classification of more than two thirds of all experimentally relevant images). Participants were also asked to name the artist of each shown image, achieving a correct response rate of 76.6% for images associated with famous artists. All further follow-up measures were identical to those described in Experiment 1.

### EEG recording and ERP pre-processing

The EEG was recorded using Ag/AgCl electrodes from 62 scalp sites according to the extended 10/20 system, referenced to the left mastoid. The sampling rate was set to 500 Hz and electrode impedance was kept below 5 kOhm. An external electrode attached below the left eye measured the electrooculogram generated from eye movements and blinks. In a short calibration procedure, prototypical eye movements were obtained to correct for ocular artifacts. Offline, the continuous EEG was re-referenced to a common average reference and band-pass filtered (low cut-off 0.01 Hz, high cut-off 40 Hz). Ocular artifacts were removed by estimating spatiotemporal dipole distributions in BESA^57^. Further artifacts (defined as segments containing amplitude values ± 200 μV or gradients > 50 μV, or containing baseline drifts) were also excluded from further analyses. The corrected EEG was then segmented into epochs, starting 200 ms prior to stimulus onset and continuing for the duration of the picture presentation. The pre-stimulus baseline was defined as 200 ms prior to picture onset. No electrodes were interpolated and 1.7% of trials were rejected across participants.

A processing pipeline focusing on single trial-based analyses using linear mixed models was implemented to analyse the EEG data^58^. ERP amplitudes were obtained by averaging across the time-windows of interest at topographical sites typically associated with the components: P1 (electrode sites: O1, O2, Oz, PO7, PO8; 110–160 ms following image onset); N1/N170 (electrode sites: TP9, TP10, P7, P8, PO9, PO10, O1, O2; 150–200 ms following image onset); EPN (electrode sites: PO7, PO8, PO9, PO10, TP9, TP10; 230–330 ms following image onset); LPP (electrode sites: Pz, Cz, C1, C2, CP1, CP2; 400–700 ms following image onset); N400: (electrode sites: C1, Cz, C2, CP1, CPz, CP2 CP2; 300–500 ms following image onset). As anticipated in the preregistration, slight adjustments were made to the preregistered time-frames on the basis of visual inspection of the spatiotemporal profiles of the components (10 ms and 30 ms for the P1 and EPN respectively).

### Primary analyses

Our primary analyses focused on the behavioural ratings and the EPN and LPP components. Due to considerations of consistency and comparability, only the first post-learning liking trial was included in the LMM analysing liking ratings. The models analysing liking, arousal and quality were therefore identical to those described in Experiment 1. As there were no pre-learning data for the LMM analyses of the EPN and LPP components and the additional behavioural variables (interest and willingness to display), time of rating was not modelled as an interactive term in these analyses. Random slopes which prevented model convergence were calculated via singular value decomposition and removed.

### Secondary analyses

As preregistered, P1, N1/N170 and N400 components were also analysed via LMMs using the same fixed and random effect structures as specified for the EPN and LPP components. We also pooled data for the dimension liking, arousal and quality from both experiments to assess the effects of valence of knowledge and artist renown on these dependent variables with an increased power. As the variables were measured on different scales (7 vs 5), the ratings were z-transformed prior to analyses^59^. A dummy variable “experiment” with two levels was created, contrast-coded and included in each model as an interactive fixed effect and as an interactive by-item and by-participant random slope. To reduce model complexity, only the after-learning ratings were analysed. Follow-up models, nesting the factor valence of knowledge in the factor artist renown, were also analysed for the ERP components and the pooled behavioural ratings. Results from the follow-up models can be found in Supplementary Material; significant differences are reported below.

### Results

#### Primary analyses

##### Behavioural ratings

Replicating the results from Experiment 1, after the learning phase, paintings presented in the negative condition were liked less (*b* =  − 0.77, 95% *CI* [− 0.96 to − 0.58], *p* < 0.001), found more arousing (*b* = 0.21, 95% *CI* [0.02 to 0.41], *p* = 0.034) and were judged qualitatively worse (*b* =  − 0.39, 95% *CI* [− 0.58 to − 0.21], *p* < 0.001), than paintings in the neutral condition. See Fig. 2b. Again, the factor artist renown did not have a significant main effect (liking: *b* = 0.09, 95% *CI* [− 0.10 to 0.28], *p* = 0.331; arousal: *b* = 0.00, 95% *CI* [− 0.27 to 0.26], *p* = 0.979; quality: *b* = 0.10, 95% *CI* [− 0.08 to 0.29], *p* = 0.267) or a modulating effect on these dimensions (liking: *b* = 0.21, 95% *CI* [− 0.31 to 0.73], *p* = 0.423; arousal: *b* = 0.01, 95% *CI* [− 0.41 to 0.42], *p* = 0.969*;* quality: *b* = 0.13, 95% *CI* [− 0.28 to 0.54], *p* = 0.535). LMMs for the two additional behavioural dependent variables revealed that participants were less willing to display a painting by an artist with a negative biography than a neutral one (*b* =  − 0.65, 95% *CI* [− 0.86 to − 0.44], *p* < 0.001) and also rated such paintings as less interesting (*b* =  − 0.22, 95% *CI* [− 0.42 to − 0.02], *p* = 0.032). See Supplementary Tables S2 and S3 for full statistical output.

##### ERPs

**Figure 3:**
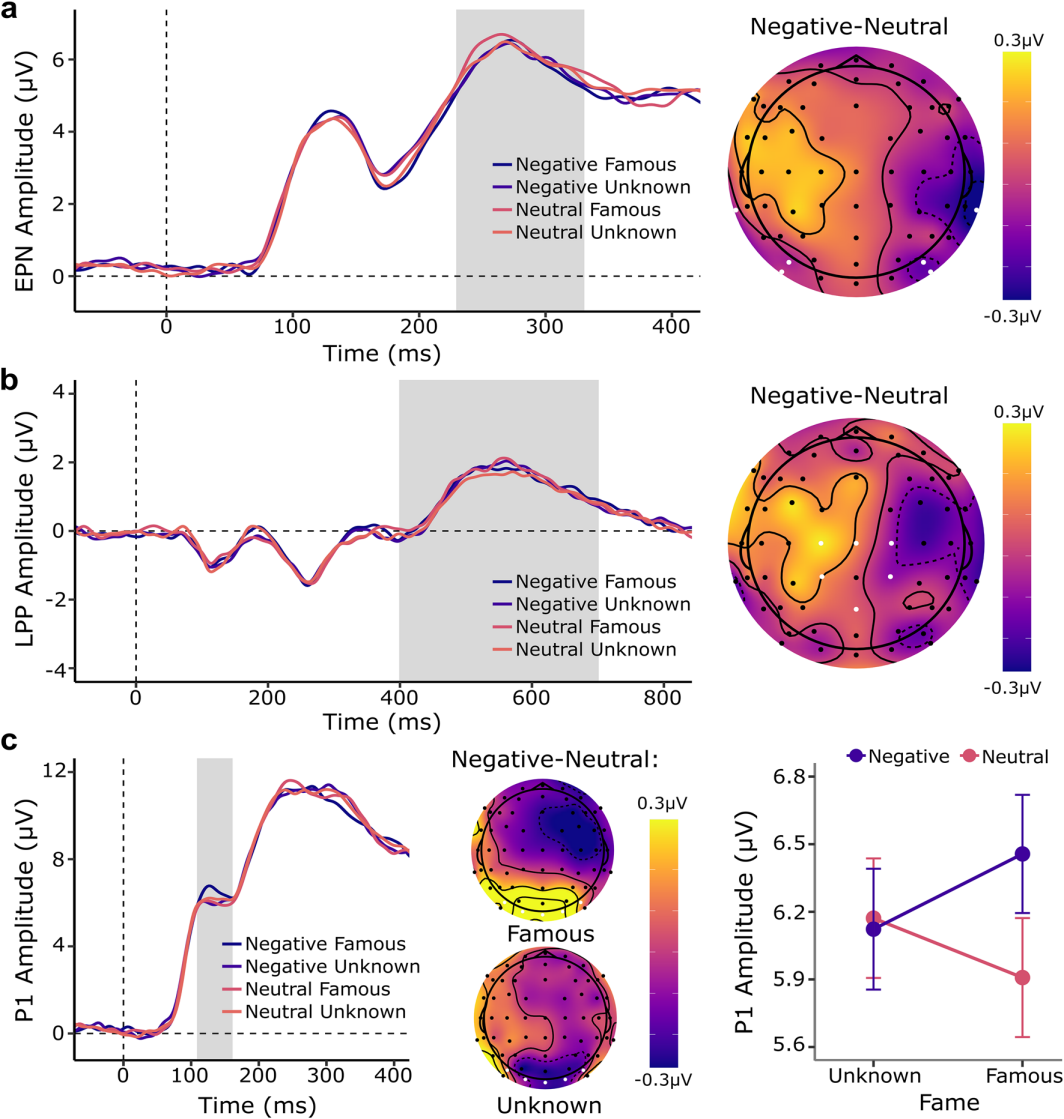
Effect of valence of biographical knowledge and artist renown on EPN, LPP and P1 amplitudes. (**a**) Grand average event-related potentials and topographical distribution at the region of interest associated with the EPN component. The difference between negative and neutral knowledge resulted in a posterior negativity between 230 and 330 ms (grey area). (**b**) Grand average event-related potentials and topographical distribution at the region of interest associated with the LPP component. The difference between negative and neutral knowledge did not reach significance between 400 and 700 ms (grey area). (**c**) Grand average event-related potentials at the region of interest associated with the P1 component. The difference between negative and neutral knowledge resulted in an enhanced P1 amplitude between 110 and 160 ms (grey area). Interaction plot and topographical distributions of negative vs neutral knowledge split by artist renown, reveal that the knowledge effect was driven by information provided about famous artists.

As shown in Fig. 3a, compared to neutral knowledge, negative knowledge about artists elicited an enhanced negativity between 230 and 330 ms in the EPN (*b* =  − 0.27, 95% *CI* [− 0.49 to − 0.06], *p* = 0.013). This effect was not modulated by artist renown (*b* =  − 0.17, 95% *CI* [− 1.07 to 0.74], *p* = 0.708), nor was a main effect of fame found (*b* = 0.04, 95% *CI* [− 0.18 to 0.26],* p* = 0.712). LPP amplitude was not significantly affected by valence of knowledge (*b* = 0.02, 95% *CI* [− 0.28 to 0.32], *p* = 0.867), artist renown (*b* = 0.09, 95% *CI* [− 0.08 to 0.26], *p* = 0.286), or the interaction between both factors (*b* =  − 0.06, 95% *CI* [− 0.63 to 0.52], *p* = 0.838). See Fig. 3b. Full statistical outputs can be found in Supplementary Table S4.

#### Secondary analyses

##### Behavioural ratings from pooled data

The effect of negative (vs neutral) knowledge on liking, arousal and quality ratings found in Experiment 1 and 2 was confirmed by the pooled analyse (See Supplementary Table S5). Crucially, there was no interaction between knowledge and experiment (liking: *b* =  − 0.10, 95% *C*I [− 0.33 to 0.14], *p* = 0.404; arousal: *b* =  − 0.09, 95% *C*I [− 0.29 to 0.10], *p* = 0.345; quality: *b* =  − 0.08, 95% *C*I [− 0.25 to 0.08], *p* = 0.319). Despite the increase in power, the effect of knowledge was not modulated by artist renown (liking: *b* = 0.08, 95% *C*I [− 0.16 to 0.32], *p* = 0.528; arousal: *b* = 0.06, 95% *C*I [− 0.16 to 0.28], *p* = 0.577; quality: *b* =  − 0.12, 95% *C*I [− 0.06 to 0.31], *p* = 0.183. The main effect of artist renown on quality ratings found in Experiment 1, reached significance (*b* = 0.10, 95% *C*I [0.02 to 0.18], *p* = 0.014), but no effect of artist renown was found for liking (*b* = 0.05, 95% *C*I [− 0.05 to 0.15], *p* = 0.305) or arousal (*b* = 0.03, 95% *C*I [− 0.07 to 0.12], *p* = 0.605). The follow − up nested models (see Supplementary Table S6) revealed numerical differences for the knowledge effect within the artist renown conditions, but the effects remained significant for both famous and unknown artists.

##### ERPs

Between 110 and 160 ms, analyses revealed a main effect of valence of knowledge on P1 amplitude (*b* = 0.25, 95% *C*I [0.03 to 0.47], *p* = 0.026). This effect was not significantly modulated by the factor artist renown (*b* = 0.61, 95% *CI* [− 0.25 to 1.47], *p* = 0.155), nor was a main effect of artist renown detected (*b* = 0.03, 95% *CI* [− 0.30 to 0.37], *p* = 0.839). Nesting the factor knowledge within the factor artist renown revealed that the knowledge effect was only significant for negative vs neutral information pertaining to famous artists (*b* = 0.56, 95% *CI* [0.08 to 1.03],* p* = 0.024), as opposed to unknown artists (*b* =  − 0.06, 95% *CI* [− 0.53 to 0.42], *p* = 0.811). This difference is illustrated in Fig. 3c. See Supplementary Tables S7 and S8 for full statistical output.

No significant effect of knowledge (*b* = 0.00, 95% *CI* [− 0.21 to 0.20], *p* = 0.976), artist renown (*b* = 0.06, 95% *C*I [− 0.31 to 0.43], *p* = 0.729), or an interaction between both factors (*b* =  − 0.38, 95% *CI* [− 1.24 to 0.48], *p* = 0.366) could be found on N1 amplitude. For the N400, analyses also did not reveal an effect of knowledge (*b* = 0.05, 95% *CI* [− 0.21 to 0.30], *p* = 0.707). The factor artist renown (*b* = 0.11, 95% *CI* [− 0.06 to 0.28], *p* = 0. 201) and the interaction term (*b* = 0.06, 95% *CI* [− 0.46 to 0.57], *p* = 0.820) did not have a significant effect on amplitude. For full statistical outputs see Supplementary Table S7. Nested models did not reveal significant differences for the factor knowledge when nested in the artist renown conditions separately for N1/N170 or N400 components (see Supplementary Table S8) or for EPN and LPP components (see Supplementary Table S9).

### Discussion

In the present study we investigated the influence of social-emotional biographical information about artists on the aesthetic experience of their paintings, whilst taking artist fame into account. We experimentally manipulated the factors valence of biographical knowledge and artist renown in two fully counterbalanced experiments, measuring a range of perceptual, emotional, and evaluative variables via behavioural ratings and electrophysiological markers of brain responses. In Experiment 1, social-emotional knowledge influenced liking, arousal and quality judgments for both famous and unknown artists. This behavioural pattern was fully replicated in the EEG experiment, which further showed that the aesthetic judgments were underpinned by enhanced brain responses associated with low-level perceptual processing (reflected in a modulation of the P1 component between 110 and 160 ms after the presentation of the painting) and early emotional processing (reflected in the EPN component between 230 and 330 ms). The results indicate that affective biographical knowledge about artists not only changes the aesthetic evaluation of an artwork and the emotional arousal associated with viewing it, but it can also penetrate early visual processes underlying the perception of the artwork itself.

In both experiments, paintings by artists who were associated with negative information were liked less and rated as more arousing than paintings by artists presented with neutral information. These findings are fully in line with those of our previous study^2^, and add evidence to the premise that affective knowledge about an artist can shape emotional responses to a painting. Unexpectedly, and in contrast to the previous study, paintings by “immoral” artists were also evaluated lower in terms of quality. The difference between our earlier and current findings can be explained by the fact that information provided in Kaube et al.^2^ referred namelessly to the presented painters as “the artist”. The resulting anonymity may have increased the perceived socio-emotional distance between artist and recipient, thereby facilitating a shift to the more detached appraisal necessary when evaluating formal qualities of an artwork “objectively”^17^. Supporting this perspective, the presence of an artist’s name can exert a top-down influence on various aesthetic judgments^60,61^ and affects the valuations of paintings in the art market^62,63^. By providing names, the present study enhanced the credibility of the information, thereby also enhancing the chances to detect effects on outcomes related to authenticity^64^. We therefore extend our previous findings by showing that knowledge of an artist’s moral transgressions can influence emotional as well as evaluation-based aesthetic outcomes^18^.

Due to the association between arousal and interest^34,35^, we expected negative, as opposed to neutral information about artists to increase interest in paintings; however, results indicated a small knowledge effect in the opposite direction (see Experiment 2). While negative stimuli can increase arousal, they can also cause disengagement^65^, especially when the moral values represented in or by the stimuli are inconsistent with one’s own^36^. For example, in the artistic realm of fiction, a disparity between the ethical beliefs held by the reader and those propounded by the work can cause the former to (un)intentionally resist further imaginative engagement with the narrative^66,67^. In the present study, the self-reported decrease in interest may thereby signify an act of rejection intended to demonstrate moral concern^68^. Negative appraisals of an artwork, in this case elicited by knowledge about its creator, also evoke hostile feelings, such as disgust^36,69^ which in turn can have behavioural implications; for example, refusing to take a postcard version of the offending picture home^36^. More generally, individuals are less willing to interact with an object if it was previously owned by someone connotated negatively, due to the well-researched contagion heuristic^37,70^. In line with this, in the present study, after being exposed to negative biographical information about an artist, participants also demonstrated a significantly reduced desire to display the paintings in their homes.

The influence of affective knowledge on aesthetic outcomes was not modulated by artist renown. Participants were selected on the basis of being highly familiar with the names of the famous artists, while the artists in the unknown condition were entirely fictitious, ensuring participants could not have been familiar with them. Therefore, the absence of a significant interaction between the factors cannot be attributed to a lack of distinction between the artist renown conditions. Instead, our findings suggest that knowing something bad about an artist influences the aesthetic judgments, regardless of whether the artist is well-known or not. A small main effect of artist renown on quality ratings (see Experiment 1) which reached significance in the pooled behavioural data model (see Experiment 2), is in accordance with literature that finds differences in artistic quality of paintings by famous vs non-famous artists^20,21^. No main effect of artist renown was found for the dimensions of liking or arousal. Participants therefore integrated information about fame into assessments of quality, but made liking and arousal judgments independently. Similarly, in research combining the experimental manipulation of presence of artist name and artist renown, results do not consistently favour the well-known artists^60^. For example, a famous artist’s work was deemed a better investment than that by a lesser-known artist, but it was not seen as more moving, more aesthetically pleasing or interesting^60^. Our findings therefore offer further evidence in support of theoretical and empirical accounts denoting the relative independence of different aesthetic outcomes^17,18,71,72^.

Replicating our original findings, negative knowledge about artists affected early brain responses associated with the automatic processing of emotional stimuli (EPN). The temporal and topographical characteristics of the modulation are comparable to those found in studies investigating the influence of affective information on object and face perception^40,42^. Our results thus indicate that when individuals are exposed to an artwork by a “bad” person, it can induce a similar emotional involvement as when they are confronted with an image of a “bad” person. Again, we did not find an effect of knowledge on LPP amplitude. Beyond a more controlled and elaborate evaluation of the emotional content of stimuli, the LPP reflects higher-order functions^73^ which are sensitive to factors such as personal and social significance^39,40,74^ and task-relevance^75,76^. Compared to the first study by Kaube et al.^2^, we increased the personal and social relevance of the information by including names and presenting famous artists that participants were familiar with. The lack of modulation for any of the conditions is therefore likely due to the task. As we aimed to explore processes which occur naturally in an interaction with an artwork, participants were instructed to answer spontaneously and were not explicitly directed to concentrate on the information about the artist when rating the paintings. The LPP is however dependent on attentional processes induced by instructions^77,78^ and participants may need to consciously attend to the acquired emotional information for affective knowledge based LPP effects to occur^78^. Moreover, evidence from the field of empirical aesthetics^79^ indicates that a lateralized late positivity between 500 and 770 ms, reflective of aesthetic appreciation, does not occur spontaneously and requires instruction. In contrast, liking, the rating task during which we recorded the EEG, is readily accessible and does not necessitate intentional contemplation^80^. To further current understanding of the complex interplay between affective knowledge and task on later processing stages involved in art evaluation, future research should consider recording EEG during multiple tasks and/or varying attention to presented content via differing instructions.

To further elucidate the cognitive dynamics involved in the processing of artworks we also explored the effect of knowledge and fame on ERP components associated with perceptual (P1 and N1/N170) and semantic processing (N400). Analyses revealed that differences between negative and neutral biographical information could be traced as early as 110 ms following stimulus presentation. As the brain activity reflected in the P1 component is linked to the perception of low-level visual features, the finding suggests that we may literally perceive pieces of art, at least in part, in light of our social-emotional knowledge about the artist. The time frame of the modulation aligns with findings from an emergent body of neuroscientific studies that have also observed a significant influence of semantic knowledge on brain activity associated with early perceptual processes^47,48,81,82^. Such empirical evidence adds credence to the possibility of the penetrability of perception via cognition, a claim subject to much philosophical debate^83–85^. Further planned analyses revealed that the knowledge effect was more pronounced for paintings associated with famous artists, suggesting that basic perceptual aspects of aesthetic experiences may be more strongly affected when we are faced with the work of a renowned artist. Correspondingly, famous faces have been shown to elicit larger P1 responses than unknown faces^86^, as have negative (i.e., angry) and socially relevant faces^74^, indicating that the effect may be driven by visual attention.

We did not find effects on N1/N170 amplitude; an electrophysiological marker of higher-level configural processing during perception. As participants in this study were non-experts, this result is consistent with research evincing the component’s functional sensitivity to expertise across multiple modalities and domains^87–89^, including art^90^. Lastly, the lack of N400 modulation aligns with previous literature which did not find the component amendable to emotional priming^91,92^. Turning to the time course of aesthetic evaluations as denoted by electrocortical correlates, research indicates that geometric patterns judged as not-beautiful, prompt a frontocentral phasic negativity around 300 to 400 ms^93^. Our results suggest that negative affective knowledge may be extracted earlier, therefore potentially in time to influence the processes underlying such judgments.

An overarching aim of the present study was to extend the generalisability of previous findings by increasing the external validity of the presented information. Whilst the same images and information were presented in Experiment 1 and 2, there were some methodological differences between the two experiments, including: settings (online vs laboratory), intensity of learning phase (information repeated once vs several times), answer scales (7 vs 5 points), response time (unlimited vs maximum of 2.5 s), structure of learning and test phases (integrated vs separate), screening mechanisms and size of images. Despite these differences, the main behavioural results of Experiment 1 were replicated in Experiment 2, indicating that the found effects are invariant to these additional influences. Further, Experiment 1 was conducted in a physical context more representative of a natural interaction with art viewed in an online environment. Recent developments in technology have rendered online settings a viable way to consume cultural objects, and the increase in virtual galleries and museum exhibitions^94,95^ confers the ecological validity of investigating aesthetic interactions on screen and in online settings^96^. Nevertheless, relocating the experimental manipulation to a real-life gallery context would provide invaluable insights into how the physical context and presentation format interact with the reported knowledge effects.

The operationalisation of the artist renown condition required participants to be familiar with the famous artists. The subsequent implemented exclusion and pre-screening mechanisms resulted in a selection of participants who were not experts, but were sufficiently acquainted with the artists to (at least) accurately identify them as painters. This implies a specific level of prior knowledge not generalisable to all people. However, as relativity of prior knowledge is inherently implied by the concept of fame itself (i.e., someone is more or less famous depending on how well and how many people know them), the effect of fame can only be studied with reference to a sample of people familiar with the investigated subject. Examining the influence of artist renown and affective biographical knowledge in a sub-population of formally trained art experts would shed further light on the interaction between these factors and expertise. It should also be noted that, to avoid priming effects, we did not collect a measure of how much participants liked the (famous) artists. As such, the present study does not address the potential modulating effect of prior preference on art perception, nor do we show how acquiring negative information affects feelings about the artists. The role of attitude towards an artist therefore also presents an intriguing line of enquiry for future research.

In conclusion, the presented findings indicate that knowing something bad about an artist affects the aesthetic appreciation of a painting, as well as the perceptual and emotional processes which underlie an aesthetic experience. Affective information about artists, particularly famous ones, elicited an enhanced brain response indicative of the visual encoding of low-level visual properties. We therefore show that negative biographical knowledge about artists not only affects consciously expressed aesthetic judgments, but can shape the actual perception of an artwork as well. Paintings by artists associated with negative information were liked less, found more arousing and evaluated as qualitatively worse that paintings by artists with a neutral biography, regardless of whether the artists were famous or unknown. Following the plethora of accusations against prominent artists in recent years, our cultural attitude towards accountability is currently in flux and the previously reigning belief that art transcends the biography of an artist is under close scrutiny^97,98^. The neurocognitive insights garnered in the present study can further understanding of the topic by contributing an empirical perspective to the debate.

### Supplementary Information

Supplementary Tables.

## Data Availability

All datasets analysed during the study are available in the Open Science Framework repository: https://osf.io/7rs3u/.
