# Supplementary material for: Art perception is affected by negative knowledge about famous and unknown artists
Source: Sci Rep. 2024 Apr 7;14:8143. doi: 10.1038/s41598-024-58697-1 (PMC10999426; doi:10.1038/s41598-024-58697-1)
Supplement: Supplementary file 1 — Supplementary Tables. [file 41598_2024_58697_MOESM1_ESM.pdf]

Art perception is affected by negative knowledge about famous and unknown artists: Hannah Kaube & Rasha Abdel Rahman

Supplementary Tables

Table S1. Effect of valence of knowledge and artist renown on liking, arousal and quality, before and after knowledge acquisition (Experiment 1).

**Predictors**	**Liking**			**Arousal**			**Quality**		
	b	*CI*	*p*	*b*	*CI*	*p*	*b*	*CI*	*p*
Intercept	3.87	3.48 – 4.26	<0.001***	3.95	3.59 – 4.31	<0.001***	5.08	4.67 – 5.49	<0.001***
Learning(Post-Pre)	-0.28	-0.45 – -0.11	0.001**	0.15	-0.01 – 0.31	0.068	-0.31	-0.47 – -0.15	<0.001***
Pre/Knowledge(Neg-Neu)	-0.09	-0.34 – 0.15	0.447	-0.07	-0.32 – 0.18	0.576	0.03	-0.15 – 0.22	0.740
Post/Knowledge(Neg-Neu)	-0.61	-0.85 – -0.37	<0.001***	0.36	0.12 – 0.61	0.004**	-0.26	-0.44 – -0.08	0.006**
Pre/Fame(F-U)	0.03	-0.21 – 0.27	0.800	-0.01	-0.30 – 0.27	0.923	-0.10	-0.35 – 0.15	0.420
Post/Fame(F-U)	0.07	-0.17 – 0.31	0.554	0.09	-0.20 – 0.38	0.544	0.23	-0.03 – 0.48	0.077
Pre/Knowledge:Fame	-0.20	-0.89 – 0.49	0.558	0.40	-0.28 – 1.09	0.240	0.01	-0.49 – 0.50	0.978
Post/Knowledge:Fame	0.05	-0.81 – 0.90	0.907	0.19	-0.48 – 0.85	0.571	0.27	-0.22 – 0.76	0.277
**Random Effects**	*Variance*	*SD*		*Variance*	*SD*		*Variance*	*SD*	
Participant									
Intercept	0.32	0.56		0.33	0.57		0.34	0.58	
Learning	\\	\\		\\	\\		0.07	0.27	
Knowledge:Fame	1.31	1.15		1.59	1.26		0.93	0.96	
Knowledge:Learnig	\\	\\		0.09	0.30		\\	\\	
Knowledge:Fame:Learning	\\	\\		0.09	0.30		\\	\\	
Item									
Intercept	0.40	0.63		0.32	0.57		0.46	0.68	
Fame	\\	\\		0.11	0.33		0.10	0.32	
Knowledge:Fame	0.52	0.72		0.21	0.46		\\	\\	
Knowledge:Learning	\\	\\		0.10	0.32		\\	\\	
Knowledge:Fame:Learning	0.77	0.88		\\	\\		\\	\\	
Residual	2.18	1.48		1.88	1.37		1.27	1.13	

*Note*s: Learning refers to the time of rating, Pre = before learning, Post = after learning. Knowledge refers to the valence of the biographical information, Neg-Neu = Negative-Neutral. Fame refers to the renown of the artist, F-U=Famous-Unknown. "/" indicates nesting of fixed factors. ":" indicates interactions between factors.

Maximal models specified: rating ~ time of rating/(knowledge*fame) + (knowledge*fame*time of rating |Participant) + (knowledge*fame time of rating |Item). Random slopes which prevented model convergence were assessed via single value decomposition and removed. “\\” indicates that the random slope was removed. *** p <.001, ** p <.01, * p <.05.

Table S2. Effect of valence of knowledge and artist renown on liking, arousal and quality, before and after knowledge acquisition (Experiment 2).

**Predictors**	**Liking**			**Arousal**			**Quality**		
	b	*CI*	*p*	*b*	*CI*	*p*	*b*	*CI*	*p*
Intercept	2.96	2.71 – 3.21	<0.001***	3.08	2.79 – 3.36	<0.001***	3.29	3.02 – 3.55	<0.001***
Learning(Post-Pre)	-0.27	-0.40 – -0.13	<0.001***	0.15	0.01 – 0.28	0.04*	-0.03	-0.16 – 0.10	0.693
Pre/Knowledge(Neg-Neu)	0.08	-0.11 – 0.27	0.427	0.00	-0.20 – 0.19	0.977	0.08	-0.10 – 0.27	0.391
Post/Knowledge(Neg-Neu)	-0.77	-0.96 – -0.58	<0.001***	0.21	0.02 – 0.41	0.034*	-0.39	-0.58 – -0.21	<0.001***
Pre/Fame(F-U)	0.00	-0.20 – 0.19	0.971	0.13	-0.13 – 0.40	0.318	0.02	-0.16 – 0.21	0.802
Post/Fame(F-U)	0.09	-0.10 – 0.28	0.331	0.00	-0.27 – 0.26	0.979	0.10	-0.08 – 0.29	0.267
Pre/Knowledge:Fame	0.12	-0.40 – 0.64	0.651	-0.24	-0.66 – 0.17	0.244	-0.07	-0.48 – 0.34	0.741
Post/Knowledge:Fame	0.21	-0.31 – 0.73	0.423	0.01	-0.41 – 0.42	0.969	0.13	-0.28 – 0.54	0.535
**Random Effects**	*Variance*	*SD*		*Variance*	*SD*		*Variance*	*SD*	
<u>Participant</u>									
Intercept	0.15	0.39		0.05	0.23		0.11	0.33	
Learning	\\	\\		\\	\\		\\	\\	
Knowledge:Fame	0.48	0.70		0.10	0.32		0.24	0.49	
Knowledge:Learning	\\	\\		\\	\\		\\	\\	
Knowledge:Fame:Learning	\\	\\		\\	\\		\\	\\	
<u>Item</u>									
Intercept	0.14	0.38		0.25	0.50		0.19	0.43	
Fame	\\	\\		0.11	0.34		\\	\\	
Knowledge:Fame	0.21	0.45		\\	\\		\\	\\	
<u>Residual</u>	1.18	1.09		1.27	1.13		1.11	1.05	

*Note*s: Learning refers to the time of rating, Pre = before learning, Post = after learning. Knowledge refers to the valence of the biographical information, Neg-Neu = Negative-Neutral. Fame refers to the renown of the artist, F-U=Famous-Unknown. "/" indicates nesting of fixed factors. ":" indicates interactions between factors.

Maximal models specified: rating ~ time of rating/(knowledge*fame) + (knowledge*fame*time of rating |Participant) + (knowledge*fame time of rating |Item). Random slopes which prevented model convergence were assessed via single value decomposition and removed. "\\" indicates that the random slope was removed. *** p <.001, ** p <.01, * p <.05.

Table S3. Effect of valence of knowledge and artist renown on interest and willingness to display (Experiment 2).

**Predictors**	**Interest**			**Willingness to display**		
	b	*CI*	*p*	*b*	*CI*	*p*
Intercept	2.99	2.70 – 3.28	<0.001***	2.41	2.10 – 2.73	<0.001***
Knowledge(Neg-Neu)	-0.22	-0.42 – -0.02	0.032*	-0.65	-0.86 – -0.44	<0.001***
Fame(F-U)	0.01	-0.19 – 0.21	0.927	0.10	-0.11 – 0.31	0.358
Knowledge:Fame	0.17	-0.23 – 0.58	0.394	0.07	-0.54 – 0.68	0.807
**Random Effects**	*Variance*	*SD*		*Variance*	*SD*	
Participant						
Intercept	0.13	0.36		0.24	0.49	
Knowledge:Fame	\\	\\		0.09	0.29	
Item						
Intercept	0.20	0.45		0.21	0.46	
Knowledge:Fame	\\	\\		0.52	0.72	
Residual	1.32	1.15		1.43	1.19	

Notes: Knowledge refers to the valence of the biographical information, Neg-Neu = Negative-Neutral. Fame refers to the renown of the artist, F-U=Famous-Unknown. ":" indicates interactions between factors.
Maximal models specified: rating ~ knowledge*fame + (knowledge*fame|Participant) + (knowledge*fame |Item).
Random slopes which prevented model convergence were assessed via single value decomposition and removed. “\\” indicates that the random slope was removed. *** p <.001, ** p <.01, * p <.05.

Table S4. Effect of valence of knowledge and artist renown on EPN and LPP amplitude (Experiment 2).

**Predictors**	**EPN (230-330ms)**			**LPP (400-700ms)**		
	b	*CI*	*p*	*b*	*CI*	*p*
Intercept	6.00	4.62 – 7.37	<0.001***	1.19	0.40 – 1.99	0.004**
Knowledge(Neg-Neu)	-0.27	-0.49 – -0.06	0.013*	0.02	-0.28 – 0.32	0.867
Fame(F-U)	0.04	-0.18 – 0.26	0.712	0.09	-0.08 – 0.26	0.286
Knowledge:Fame	-0.17	-1.07 – 0.74	0.708	-0.06	-0.63 – 0.52	0.838
**Random Effects**	*Variance*	*SD*		*Variance*	*SD*	
Participant						
Intercept	10.78	3.28		3.84	1.96	
Knowledge:Fame	1.81	1.35		0.53	0.73	
Item						
Intercept	1.98	1.41		0.54	0.73	
Knowledge	\\	\\		0.19	0.44	
Knowledge:Fame	1.36	1.17		0.45	0.67	
Residual	24.54	4.95		15.12	3.89	

Notes: Knowledge refers to the valence of the biographical information, Neg-Neu = Negative-Neutral. Fame refers to the renown of the artist, F-U=Famous-Unknown. ":" indicates interactions between factors.
Maximal models specified: ERP ~ knowledge*fame + (knowledge*fame |Participant) + (knowledge*fame |Item).
Random slopes which prevented model convergence were assessed via single value decomposition and removed.
"\\" indicates that the random slope was removed. *** p <.001, ** p <.01, * p <.05.

Table S5. Analyses of valence of knowledge and artist renown on liking, arousal and quality for data pooled across Experiment 1 and Experiment 2.

**Predictors**	**Liking**			**Arousal**			**Quality**		
	b	*CI*	*p*	*b*	*CI*	*p*	*b*	*CI*	*p*
Intercept	-0.24	-0.42 – -0.07	0.008**	-0.05	-0.23 – 0.13	0.545	0.26	0.06 – 0.46	0.014*
Knowledge(Neg-Neu)	-0.42	-0.54 – -0.31	<0.001***	0.18	0.08 – 0.27	<0.001***	-0.20	-0.28 – -0.12	<0.001***
Fame(F-U)	0.05	-0.05 – 0.15	0.305	0.03	-0.07 – 0.12	0.605	0.10	0.02 – 0.18	0.014*
Exp(2-1)	-0.56	-0.73 – -0.39	<0.001***	-0.54	-0.71 – -0.37	<0.001***	-1.01	-1.22 – -0.81	<0.001***
Knowledge:Fame	0.08	-0.16 – 0.32	0.528	0.06	-0.16 – 0.28	0.577	0.12	-0.06 – 0.31	0.183
Knowledge:Exp	-0.10	-0.33 – 0.14	0.404	-0.09	-0.29 – 0.10	0.345	-0.08	-0.25 – 0.08	0.319
Fame:Exp	0.01	-0.32 – 0.35	0.941	-0.06	-0.30 – 0.19	0.630	-0.07	-0.36 – 0.21	0.583
Knowledge:Fame:Exp	0.09	-0.39 – 0.57	0.700	-0.11	-0.54 – 0.33	0.619	-0.09	-0.45 – 0.28	0.642
**Random Effects**	*Variance*	*SD*		*Variance*	*SD*		*Variance*	*SD*	
Participant									
Intercept	0.08	0.29		0.08	0.28		0.09	0.30	
Knowledge:Exp	0.27	0.52		\\	\\		\\	\\	
Knowledge:Fame:Exp	1.30	1.14		0.59	0.77		0.42	0.65	
Item									
Intercept	0.08	0.29		0.09	0.30		0.12	0.35	
Exp	\\	\\		\\	\\		0.05	0.23	
Fame:Exp	0.23	0.48		0.22	0.22		0.17	0.41	
Residual	0.65	0.81		0.65	0.81		0.46	0.68	

*Note*s: Knowledge refers to the valence of the biographical information, Neg-Neu = Negative-Neutral. Fame refers to the renown of the artist, F-U=Famous-Unknown. Experiment is a dummy coded control variable indicating which experiment the data is from, 2-1 = Experiment 2 – Experiment1 ":" indicates interactions between factors.

Maximal models specified were: rating ~ knowledge*fame*exp + (knowledge*fame*exp|Participant) + (knowledge*fame*exp |Item).

Random slopes which prevented model convergence were assessed via single value decomposition and removed. “\\” indicates that the random slope was removed. *** p <.001, ** p <.01, * p <.05.

Table S6. Effect of valence of knowledge nested in artist renown on liking, arousal and quality for data pooled across Experiment 1 and Experiment 2.

**Predictors**	**Liking**			**Arousal**			**Quality**		
	b	*CI*	*p*	*b*	*CI*	*p*	*b*	*CI*	*p*
(Intercept)	-0.24	-0.42 – -0.07	0.008**	-0.05	-0.23 – 0.13	0.545	0.26	0.06 – 0.46	0.014
Fame(F-U)	0.05	-0.05 – 0.15	0.305	0.03	-0.07 – 0.12	0.605	0.1	0.02 – 0.18	0.014
U/Knowledge(Neg-Neu)	-0.46	-0.62 – -0.30	<0.001***	0.15	0.00 – 0.29	0.046*	-0.26	-0.39 – -0.14	<0.001***
F/Knowledge(Neg-Neu)	-0.38	-0.56 – -0.21	<0.001***	0.21	0.06 – 0.35	0.005**	-0.14	-0.26 – -0.02	0.025*
U/Exp(2-1)	-0.57	-0.80 – -0.33	<0.001***	-0.51	-0.71 – -0.31	<0.001***	-0.97	-1.21 – -0.74	<0.001***
F/Exp(2-1)	-0.55	-0.79 – -0.32	<0.001***	-0.57	-0.77 – -0.36	<0.001***	-1.05	-1.31 – -0.79	<0.001*
U/Knowedge:Exp	-0.15	-0.47 – 0.18	0.369	-0.04	-0.33 – 0.25	0.793	-0.04	-0.28 – 0.20	0.749
F/Knowedge:Exp	-0.05	-0.40 – 0.30	0.768	-0.15	-0.44 – 0.14	0.316	-0.13	-0.37 – 0.12	0.311

*Note*s: Knowledge refers to the valence of the biographical information, Neg-Neu = Negative-Neutral. Fame refers to the renown of the artist, F-U=Famous-Unknown. Experiment is a dummy coded control variable indicating which experiment the data is from, 2-1 = Experiment 2 – Experiment1. ":" indicates interactions between factors. "/" indicates nesting of fixed factors. Random effects are identical to those presented in Table S5.
*** p <.001, ** p <.01, * p <.05.

Table S7. Analyses of valence of knowledge and artist fame on P1, N1/N170 and N400 amplitude (Experiment 2).

**Predictors**	**P1 (110-160ms)**			**N1/N170 (150-200ms)**			**N400 (300-500ms)**		
	b	*CI*	*p*	*b*	*CI*	*p*	*b*	*CI*	*p*
Intercept	6.15	4.16 – 8.14	<0.001***	3.33	1.60 – 5.06	<0.001***	-0.38	-1.26 – 0.51	0.393
Knowledge(Neg-Neu)	0.25	0.03 – 0.47	0.026*	0.00	-0.21 – 0.20	0.976	0.05	-0.21 – 0.30	0.707
Fame(F-U)	0.03	-0.30 – 0.37	0.839	0.06	-0.31 – 0.43	0.729	0.11	-0.06 – 0.28	0.201
Knowledge:Fame	0.61	-0.25 – 1.47	0.155	-0.38	-1.24 – 0.48	0.366	0.06	-0.46 – 0.57	0.820
**Random Effects**	*Variance*	*SD*		*Variance*	*SD*		*Variance*	*SD*	
Participant									
Intercept	26.22	5.12		11.45	3.38		4.84	2.20	
Fame	0.46	0.68		0.11	0.34		\\	\\	
Knowledge:Fame	2.12	1.46		0.69	0.83		\\	\\	
Item									
Intercept	2.42	1.56		5.93	2.44		0.62	0.79	
Knowledge	\\	\\		\\	\\		0.10	0.32	
Fame	\\	\\		0.26	0.51		\\	\\	
Knowledge:Fame	0.91	0.96		1.65	1.28		0.44	0.66	
Residual	25.07	5.01		21.77	4.67		15.37	3.92	

Notes: Knowledge refers to the valence of the biographical information, Neg-Neu = Negative-Neutral. Fame refers to the renown of the artist, F-U=Famous-Unknown. ":" indicates interactions between factors.

Maximal models specified: ERP ~ knowledge*fame + (knowledge*fame|Participant) + (knowledge*fame |Item).

Random slopes which prevented model convergence were assessed via single value decomposition and removed. “\\” indicates that the random slope was removed. *** p <.001, ** p <.01, * p <.05.

Table S8. Effect of valence of knowledge nested in factor artist renown on P1, N1/N170 and N400 amplitude (Experiment 2)

**Predictors**	**P1 (110-160ms)**			**N1/N170 (150-200ms)**			**N400 (300-500ms)**		
	b	*CI*	*p*	*b*	*CI*	*p*	*b*	*CI*	*p*
Intercept	6.15	4.16 – 8.15	<0.001***	3.33	1.60 – 5.06	<0.001***	-0.38	-1.26 – 0.51	0.393
Fame(F-U)	0.03	-0.30 – 0.37	0.839	0.06	-0.29 – 0.41	0.712	0.11	-0.06 – 0.28	0.201
U/Knowledge(Neg-Neu)	-0.06	-0.53 – 0.42	0.811	0.19	-0.31 – 0.68	0.437	0.02	-0.38 – 0.42	0.927
F/Knowledge(Neg-Neu)	0.56	0.08 – 1.03	0.024*	-0.19	-0.70 – 0.31	0.430	0.07	-0.25 – 0.40	0.632

Notes: Knowledge refers to the valence of the biographical information, Neg-Neu = Negative-Neutral. Fame refers to the renown of the artist, F-U=Famous-Unknown. "/" indicates nesting of fixed factors. Random effects are identical to those presented in Table S7.
*** p <.001, ** p <.01, * p <.05.

Table S9. Effect of valence of knowledge nested in factor artist renown on EPN (230-330ms) and LPP (400-700ms) (Experiment 2)

**Predictors**	**EPN (230-330ms)**			**LPP (400-700ms)**		
	b	*CI*	*p*	*b*	*CI*	*p*
Intercept	6.00	4.62 – 7.37	<0.001***	1.19	0.40 – 1.99	0.004**
Fame(F-U)	0.04	-0.18 – 0.26	0.712	0.09	-0.08 – 0.26	0.286
U/Knowledge(Neg-Neu)	-0.19	-0.69 – 0.30	0.437	0.05	-0.42 – 0.52	0.818
F/Knowledge(Neg-Neu)	-0.36	-0.85 – 0.14	0.152	0.00	-0.35 – 0.34	0.978

Notes: Knowledge refers to the valence of the biographical information, Neg-Neu = Negative-Neutral. Fame refers to the renown of the artist, F-U=Famous-Unknown. “/” indicates nesting of fixed factors. Random effects are identical to those presented in Table S4.

*** p <.001, ** p <.01, * p <.05.
